# Data-Driven Construction Safety Information Sharing System Based on Linked Data, Ontologies, and Knowledge Graph Technologies

**DOI:** 10.3390/ijerph19020794

**Published:** 2022-01-11

**Authors:** Akeem Pedro, Anh-Tuan Pham-Hang, Phong Thanh Nguyen, Hai Chien Pham

**Affiliations:** 1 Center for Systems Engineering and Innovation, Imperial College London, London SW7 2BX, UK; lanrepedro3@gmail.com; 2 School of Computer Science and Engineering, International University, Ho Chi Minh City 700000, Vietnam; anhtuanphamhang@gmail.com; 3 Department of Project Management, Ho Chi Minh City Open University, Ho Chi Minh City 700000, Vietnam; phong.nt@ou.edu.vn; 4 Applied Computational Civil and Structural Engineering Research Group, Faculty of Civil Engineering, Ton Duc Thang University, Ho Chi Minh City 700000, Vietnam

**Keywords:** construction safety, information sharing, knowledge graph, linked data, ontology, semantic web, data-driven, knowledge engineering, knowledge management, accident prevention

## Abstract

Accident, injury, and fatality rates remain disproportionately high in the construction industry. Information from past mishaps provides an opportunity to acquire insights, gather lessons learned, and systematically improve safety outcomes. Advances in data science and industry 4.0 present new unprecedented opportunities for the industry to leverage, share, and reuse safety information more efficiently. However, potential benefits of information sharing are missed due to accident data being inconsistently formatted, non-machine-readable, and inaccessible. Hence, learning opportunities and insights cannot be captured and disseminated to proactively prevent accidents. To address these issues, a novel information sharing system is proposed utilizing linked data, ontologies, and knowledge graph technologies. An ontological approach is developed to semantically model safety information and formalize knowledge pertaining to accident cases. A multi-algorithmic approach is developed for automatically processing and converting accident case data to a resource description framework (RDF), and the SPARQL protocol is deployed to enable query functionalities. Trials and test scenarios utilizing a dataset of 200 real accident cases confirm the effectiveness and efficiency of the system in improving information access, retrieval, and reusability. The proposed development facilitates a new “open” information sharing paradigm with major implications for industry 4.0 and data-driven applications in construction safety management.

## 1. Introduction

Construction is one of the largest industries which plays a prominent role in driving economic growth and development on a global scale. The construction industry accounts for 13% of the global GDP, and this figure is projected to rise over the next decade [1]. Despite the industry’s economic significance, it remains extremely dangerous and fraught with injuries and fatalities [2,3,4,5]. Aside from the financial impact of cost overruns and time delays resulting from accidents, the humanitarian and societal cost of continuous loss of life in the industry is unquantifiable and non-negligible. Concerted efforts have been made in enforcing safety regulations, improving training initiatives, and incorporating advanced technologies and preventative protocols to improve safety performance [6,7,8,9]. However, accident rates remain high, and the industry is plagued by casualty rates as much as three times higher than those in other industries [10,11,12,13]. 

Undoubtedly, past accident data are a rich source of knowledge that should be incorporated into safety management processes to transfer lessons learned, and progressively improve safety outcomes [14]. However, these data tend to remain inaccessible and underutilized in practice. One of the factors this can be attributed to is the lack of formal standards and schemas for publishing accident data in a consistent manner. Accident data are typically unstructured and exist in a variety of documents in diverse non-machine-readable formats [15]. Knowledge embedded in such documents is unconsolidated and difficult to search semantically, and thus cannot be utilized to enrich safety management processes. As such, safety management workflows fail to benefit from data-driven information sharing applications which effectively share lessons learned and generate insights to prevent accident reoccurrence. 

Advances in knowledge engineering, information science, and computing present new unprecedented opportunities for the industry to leverage, share, and reuse safety information more efficiently. Linked data, ontologies, and knowledge graph technologies have been applied to improve knowledge management and dissemination in various industries. Linked data are defined a set of best practices for expressing, publishing, and connecting structured data by using semantic web technologies, and ontologies are complementary tools which serve as a foundation for linked data by allowing the modeling of concepts with semantic relationships [16]. Noteworthy applications of linked data can be found in governance, where the concept of linked open government data has been considered to support information transparency and innovative service development in various countries [17,18,19]. Similarly, the healthcare domain has also leveraged and benefited from linked data. For instance, open medical data have been explored to improve knowledge dissemination and diagnostics in healthcare services [20,21]. Semantic web- and graph-based technologies have also garnered some attention in construction research, however, scholarly efforts barely exploit these potentials for safety information sharing. Creating accessible mechanisms and technical infrastructures to link knowledge sources with safety processes would be of tremendous value in the construction industry, however, such systems are severely lacking in research and practice alike. 

To address this deficiency, this research develops a novel data-driven construction safety information sharing system based on linked data, ontologies, and knowledge graph technologies. The proposed development comprises the three modules. Firstly, an ontology module is developed to formalize knowledge pertaining to accident data, and in doing so, make the sharing and exchange of safety information easier. Secondly, a data processing module is designed to automatically convert existing and newly input accident data to resource description framework (RDF) format, which serves as a consistent machine-readable format for accident data representations. Lastly, a query module is developed to enable efficient and effective retrieval of safety information. Knowledge graph-based approaches are also utilized to enable visual interaction with accident data and to validate the proposed system. By leveraging advances in linked data, ontologies, and knowledge graph technologies, the developed system aims to facilitate the open dissemination and utilization of safety information from accident data. The proposed approach would support practitioners in effectively and efficiently retrieving relevant safety information to learn from past incidents, thus enabling the dissemination of data-driven insights which would contribute to accident prevention efforts in safety management processes.

The paper is structured as follows. Section 2 presents a literature review investigating the barriers and challenges to sharing construction safety information. Related works on linked data, ontologies, and knowledge graph technologies in architecture, engineering, and construction (AEC) contexts are also discussed. Section 3 presents the materials and methods of the study, describing the development of the constituent system modules. Subsequently, Section 4 addresses the evaluation and validation of the proposed solution. To conclude the paper, Section 5 and Section 6 present the discussion and conclusions, respectively.

## 2. Literature Review

Although many approaches have emerged to improve safety outcomes, very little attention has been paid to the sharing of safety information in AEC domains. Construction is a complex, information-intensive, highly interdependent process where success often depends on timely access to actionable information. The sharing of safety information is of huge significance in safety management processes and accident prevention efforts [22], however, there are numerous barriers impeding effective dissemination in practice. This section expounds on the technical and organizational challenges to safety information sharing, and then reviews works on knowledge engineering technologies such as linked data, ontologies, and knowledge graphs which can contribute to resolving the identified issues. Subsequently, the need for the proposed data-driven construction safety information system is discussed.

### 2.1. Barriers and Challenges to Safety Information Sharing in Construction

The importance of sharing information to learn from past failures is well established in safety literature [23,24]. In construction, an emphasis is placed on learning from case studies of catastrophic events, while there are few opportunities to learn from the more frequent, lower consequence failures which occur repeatedly [25]. Some corporations maintain in-house records and databases which provide access to past accident data with lessons learned. While such internal systems may provide beneficial information, they tend to be restricted to activities within the organization’s scope and such lessons learned from an organization’s projects cannot be shared with other companies. Similarly, lessons learned from external related projects cannot be acquired.

Within organizations, past mishap investigations and analyses produce a plethora of diverse outputs ranging from narrative reports, tables, and checklists to statistical trends, recommendation documents, and case books. However, there are no standardized methods for reporting on accidents [26]. Hence, documents containing accident data are generated and stored in a myriad of formats such as PDFs, CSVs, and Excel spreadsheets. These data are typically dispersed in organizational systems in unstructured, unconsolidated, and non-machine-readable representations. This problem is further exacerbated by the fact that documents with accident data are commonly published in natural language. As a result, significant time and efforts are required in retrieving the knowledge users need [27]. Moreover, traditional keyword-based searches have numerous limitations which often inhibit knowledge discovery [16]. To share the knowledge and information embedded in safety documents, mechanisms for semantically understanding data are necessary. However, developments in this regard are severely deficient in both industry efforts and research. In addition, due to the lack of formalized domain knowledge, information from accident data cannot be automatically connected to beneficial information from existing, readily available resources on the web.

Furthermore, detailed information describing the contexts around accident cases is often lacking. As pointed out by Gibb [28], it is crucial to go in depth into accidents and identify underlying causes for good learning outcomes. However, there is no universal, commonly agreed upon checklist or structure which delineates the information requirements to appropriately describe accident data. For instance, useful information on the equipment, workspaces, site conditions, and potential accident causes due to human and environmental factors is often not explicitly stated in reports. Hence, the quality, richness, and usability of past reports vary [26]. This limits the amount of learning and benefits which can be gained from past accident data. Another significant issue lies in the representation of accident data. Conventional accident data representations are based on tabular formats with spreadsheets. While these representations may provide access to huge amounts of information, they do not provide an intuitive sense of the data, its properties, shape, and distribution. These traditional approaches are not visually optimal for detecting patterns in data and communicating and conveying insights to a wide array of industry practitioners. Moreover, manually navigating through data can time-consuming, and keyword searches have numerous drawbacks in information retrieval [29].

The aforementioned conditions pose major impediments to the sharing and retrieval of beneficial safety information spanning beyond organizational boundaries. As a result, data-driven insights cannot be acquired to enhance safety management workflows and proactively prevent accident reoccurrence. Accident data are of immense value in safety planning, training, and management, and the industry continues to miss out on the potential benefits of utilizing information from past cases. A few studies have proposed approaches to improve safety information sharing. For instance, Su et al. [30] developed a case-based reasoning model for case retrieval and reuse, towards accident prevention and improved decision making. Le et al. [31] developed a social network system for sharing construction health and safety knowledge. Li et al. [22] investigated the use of web 2.0, IoT, and mobile applications in safety knowledge sharing. The study highlighted the lack of scholarly research on knowledge management and sharing in construction. However, studies thus far have failed to address the technical and organizational barriers impeding the effective and efficient dissemination of safety information in practice.

### 2.2. Ontologies, Linked Data, and Knowledge Graph-Based Approaches for Information Sharing

Over the past few decades, diverse information science techniques have been developed to improve knowledge management and dissemination in various disciplines. Ontologies have emerged as an effective tool in addressing ambiguity and consistency issues in domain-specific knowledge sharing and reuse [32]. In essence, ontologies facilitate effective dissemination and information exchange through the explicit specification of concepts, attributes, and relationships. By providing consistent structures and semantics, they can be utilized to ensure the validity of information to be communicated. To date, the W3C Web Ontology Language (OWL) is one of the most pervasive computational logic-based information modeling languages with applications and use cases spanning across diverse industries.

In construction, several ontologies have been deployed to improve information sharing and address interoperability issues. Prominent ones include the building topology ontology (BOT) which describes the core topological concepts of a building, and ifcOWL which provides an OWL representation of the industry foundation classes (IFC) open standard for representing building and construction data. Furthermore, a few studies have utilized ontologies to address information retrieval and knowledge management challenges in AEC research. For instance, Wu et al. [33] proposed an ontological approach for metro accident case retrieval through case-based reasoning and natural language processing. Guo and Yang [34] developed an ontology which formalized knowledge on active fall protection systems to facilitate knowledge sharing and reuse, and Zhang et al. [35] proposed ontology-based modeling of construction safety knowledge to enable automated safety planning for job hazard analysis (JHA) within BIM environments. Similarly, Lu et al. [36] proposed ontology-based knowledge modeling for automated construction safety checking, however, their approach leveraged the Semantic Web Rule Language and JESS rule engine. Several studies have emphasized the potentials of ontologies in effectively linking information models with safety operations. However, despite these developments, there is a lack of scholarly endeavors focused on improving the retrieval and reuse of information from past accidents.

**Figure 1:**
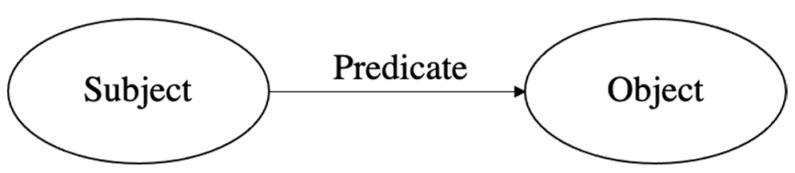
Basic RDF triple.

Linked data are a core element of the semantic web, which is an extension of the syntactic web proposed by the World Wide Web Consortium (W3C). Under the umbrella of the semantic web, linked data provide a publishing paradigm whereby the web is used to create typed links between data from different sources. This enables the machine-based exploration of data for effective sharing and reuse between applications, corporations, and communities. As set out by Berners-Lee [37], linked data are based on four basic principles: (1) the use of URIs as names for things, (2) the use of HTTP URIs so that people can look up those names, (3) the provision of useful information using the standards (RDF, SPARQL) when someone looks up a URI, and (4) the inclusion of links to other URIs so that more useful resources can be discovered. The RDF data model constitutes the fundamental building block for linked data. As illustrated in Figure 1, RDF encodes semantic relationships as triples (positional statements), comprising subjects, predicates, and objects. These triples provide a flexible and consistent way to break down complex knowledge and facilitate computational interpretation of data.

Linked data leverage the coalescence of complementary semantic web technologies such as RDF, OWL, and SPARQL to not only facilitate the encoding of semantics into web data, but also enable querying and drawing of inferences from pre-defined vocabularies. In addition, building on the foundations of the semantic web, knowledge graph approaches have also gained popularity in the past decade. In line with linked data approaches, knowledge graphs organize and integrate data according to ontology schema and enable the generation of data-driven analytics and insights. These technologies have already garnered significant attention in healthcare and medical domains [38]. Moreover, based on the Gartner hype cycle [39], their adoption along with AI is envisioned to facilitate intelligent services and propagate digital transformation in various industries.

In AEC contexts, linked data and graph-based technologies can boost digitalization and knowledge creation [40], and a few studies have explored these potentials. Research efforts have developed semantic web- and graph-based applications to improve processes in quality management and defect prevention [16], construction procurement [41], asset management [42], building energy performance management [43], knowledge management [44], and look-ahead planning [45]. While linked data have been used to improve interoperability and link across domains in AEC research [46], scholarly efforts have barely delved into the potentials for construction safety information sharing. From a broader safety research perspective, Benner [14] expounded on the challenges in the documentation, dissemination, and utilization of lessons learned from mishap investigations. In response, the study explored the idea of a semantic web-based lesson sharing system. Similarly, Batres et al. [47] proposed the use of ontologies to enhance the utilization of accident information. However, rather than sharing lessons learned, the paper developed an automated reasoning approach to capture and locate accident descriptions.

### 2.3. Need for Linked Data and Semantic Web Approaches for Safety Information Sharing

The sharing of information and lessons learned from past incidents is critical in improving safety outcomes in the construction industry. Various interventions are necessary to address the technical and organizational challenges that impede the dissemination of knowledge from past mishaps. Firstly, there is a need for common data models and formats to enable the structured and consistent publication of accident data. As emphasized by Wasilkiewicz [26], tools for accident investigation should be designed to ensure construction personnel can acquire knowledge from past events. In this regard, tools which formalize accident data descriptions and safety domain knowledge are necessary to improve the utilization and reusability of safety information. Secondly, there is a need for technical infrastructures for the integration and dissemination of accident data across disparate organizational silos. Machine-readability in accident data representations is also necessary to enable advanced information retrieval functionalities. Furthermore, rather than traditional tabular representations, scholarly efforts exploring flexible and more visually intuitive approaches for interacting with safety data and information are needed. While tools such as relational databases could address some of the identified issues with sharing safety information, there is a need for intelligent future-oriented solutions which not only support larger scale inter-organizational dissemination, but also connect and exploit the tremendous volumes of resources and information on the web.

Linked data, ontologies, and knowledge graphs have emerged as powerful tools with immense potential for digital transformation. While numerous studies have explored the potentials of these knowledge engineering technologies for data-driven information retrieval and analytics, research efforts have not developed solutions to the challenges in construction safety workflows. Based on this status quo, the construction industry urgently needs to exploit advances in ontologies, linked data, and knowledge graph technologies to resolve the pervasive issues in sharing construction safety information from past accidents. In response, this research presents a novel data-driven construction safety information sharing system based on linked data, ontologies, and knowledge graph technologies.

## 3. Materials and Methods

**Figure 2:**
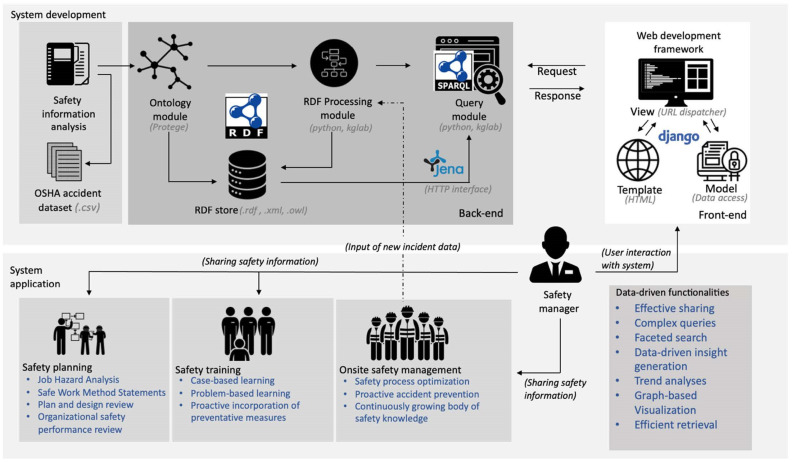
Development and application framework for construction safety information sharing system.

This study utilizes a sequential top-down protocol in developing a novel data-driven construction safety information sharing system. Figure 2 presents a development framework to illustrate the key processes and constituents in the system. The upper half of the figure depicts the technical developments, while the lower half highlights applications in safety management processes. The study commences with an information analysis of accident case data, which forms the basis for the developed system. This is followed by the back-end development comprising the following three modules:

1. Ontology module: The ontology module is designed to formalize the expertise and knowledge that goes into accident data descriptions, to ensure not only the consistent structure in data representations, but the quality and richness of descriptions as well. As illustrated in Figure 2, this module enables the formatting and saving of safety data as RDF.
2. RDF processing module: The RDF processing module is designed to facilitate the automatic conversion of newly input data and existing tabular accident data into RDF graphs through algorithms to enable information retrieval and reusability. Data handled in this module are also saved in an RDF store.
3. Query module: The query module provides access to the formalized and converted data from the ontology and RDF processing modules in the RDF store. The SPARQL Protocol and RDF Query Language (SPARQL) are deployed to facilitate advanced information retrieval functionalities, while the KGLAB [48] package enables graph-based visualization of accident data.

Trials and test scenarios are conducted with real accident cases to assess the effectiveness and efficiency of the system modules in improving information access, retrieval, and reusability. In addition to the development of the three modules, the framework includes front-end developments to enable construction personnel to access system functionalities to conveniently retrieve and share safety information. In this regard, the Django web development framework [49] is proposed with the model–template–view architectural pattern, to design and manage user interfaces and system interactions. Apache Jena Fuseki [50] is also proposed as an open-source semantic web framework which would function as a server and HTTP interface to RDF accident data. 

As portrayed in the lower half of Figure 2, the data-driven construction information sharing system is designed to enable safety managers to execute complex queries and faceted searches to acquire relevant insights from related incidents and facilitate the dissemination of lessons learned with diverse participants through the project lifecycle. From the early design stages, the proposed solution is envisioned to enrich design reviews and job hazard analyses, and aid in preparing safe work method statements during safety planning. Furthermore, the system is designed to create opportunities for case-based and problem-based safety training initiatives, allowing workers to gain context-specific knowledge based on highly related past cases. Following the description of the accident data analysis, the procedures and methods in developing the ontology and RDF processing modules are delineated in the subsequent sections. 

### 3.1. Accident Data Analysis

An analysis of construction accident data was conducted to not only shed light on accident causation factors based on recent data but also to investigate the categories, properties, and structure of accident data in a typical storage format. The analysis explored what beneficial information and knowledge can be derived from data, which subsequently guided the development of an ontology, as presented in Section 3.2. The analysis was based on a USA Occupational Health and Safety Administration (OSHA) dataset comprising 4847 accident cases from 2015–2017. OSHA is a credible leading authority on occupational health and safety and the selected dataset provided an up-to-date reflection of safety performance in the construction industry. The dataset was rich and multi-dimensional, comprising numerous detailed categorical features from which beneficial insights could be generated.

**Figure 3:**
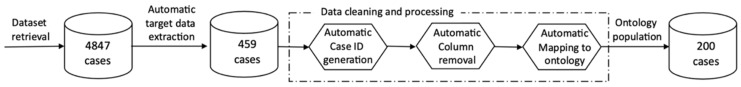
Data extraction and cleaning procedures.

As illustrated in Figure 3, a series of steps were taken in filtering and processing the data. The retrieved OSHA dataset included 4847 accident cases from construction, manufacturing, infrastructure, and other industries. In order to limit to scope of the study, cases which pertained specifically to construction were extracted automatically using a Python script with the Pandas data analysis and manipulation tool. This yielded 459 accident cases. Next, a series of scripts were written to automatically generate unique IDs for the extracted cases. The categories in the dataset were automatically mapped to align with the intended ontology categories. The data cleaning process involved removing all unrequired columns and then identifying and eliminating anomalies in the data, e.g., repeated cases, cases with errors, and cases with critical data inputs missing. The refined construction accident dataset comprising 459 cases was then analyzed, and 200 accident cases were selected to populate the developed system.

Table 1 highlights the leading accident causes based on human and environmental factors in the OSHA data. Addressing human accident causation factors requires a variety of human-centric, behavior-focused interventions in training, jobsite monitoring, etc. On the other hand, addressing environmental accident causation factors requires diverse mitigative efforts and strategies in work planning, safety engineering, etc. However, the analysis reveals that these prominent factors cause accidents repeatedly without being rectified. For instance, hazardous situations are commonly misjudged in as many as 30% of cases, and facility layout conditions are not optimized in advance to mitigate jobsite risks (22%). Similarly, material and equipment handling methods are often inappropriate and tasks are not planned or structured with adequate preventative measures (16%). In this regard, it is necessary learn from past data, proactively instill insights on accident causation, and make them available to practitioners across various safety management processes. Among the 459 construction cases in the OSHA data, 57% were fatal. These repeated work-related accidents result in losses not only to employers, but also to workers, their families, and society. It is paramount that efforts are made to share information from past mishaps to improve safety outcomes in the construction industry.

### 3.2. Ontology Module Development

The development of the ontology module was guided by the Linked Open Terms (LOT) methodology for developing ontologies and vocabularies. The methodology encourages the reuse of existing vocabularies or ontologies, however, since no vocabularies covering the accident data domain were found in registries such as Linked Open Vocabularies (LOV), a new ontology was developed in accordance with the linked data principles. Based on the LOT ontology design workflow, the development of the module included ontology requirement specification, ontology conceptualization, ontology encoding, ontology validation, and, lastly, publication.

**Figure 4:**
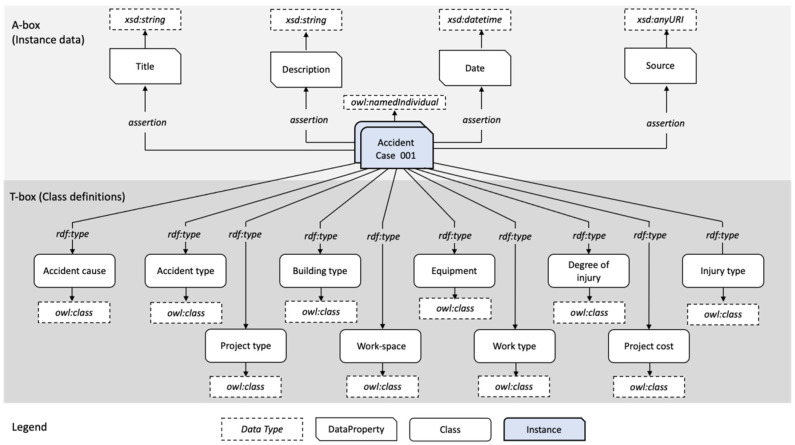
Ontology schema for accident data representations.

Based on the accident data analysis, the ontology requirements were articulated, and an ontology schema was formalized to model accident data in a unified format. The schema was designed to enable consistent data representations, and in doing so, to enable efficient acquisition of data-driven insights from accident data. As illustrated in Figure 4, the schema was designed with assertional (instance data) A-box and T-box (class definition) data. The OWL, RDF, and RDFS data modeling languages were used as they provide rich expressivity with RDF data. For instance, the owl:disjointwith relationship was used to model the disjoint relationship between fatal and non-fatal accident classes. Accident cases were modeled as named individuals (instances), with four asserted data properties, namely title, description, date, and source. The data type for the title and description properties are strings, specified through the XSD schema, while the date property is specified with an XSD schema datetime value. The source data property is assigned an anyURI value. In terms of class definitions, 7 classes were created, namely accident type, accident cause, building type, injury type, degree of injury, project type, and project cost, based on the OSHA categories. An additional 3 classes were added, namely workspace, work type, and equipment, to enrich the ontology and enable richer descriptions and broader knowledge discovery capabilities. The classes are further broken down into subclasses, for instance, the accident cause class comprises human factor and environmental factor causes as subclasses, with each comprising specific accident causes.

**Figure 5:**
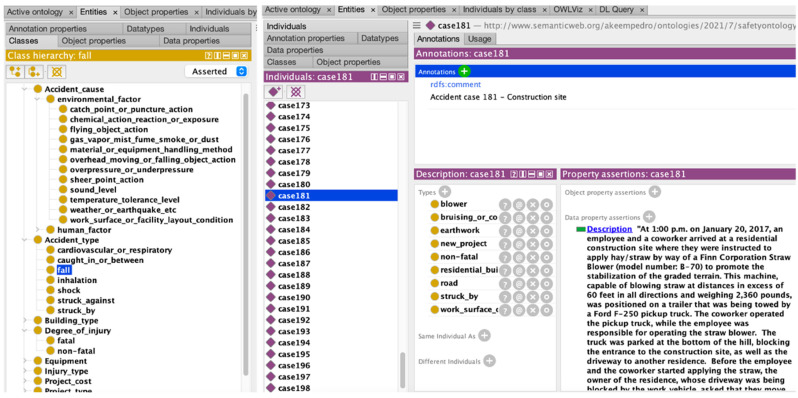
Ontology for construction safety information sharing.

As illustrated in Figure 5, the Protégé ontology editing tool was utilized to encode the ontology. Protégé is a powerful, free, open-source editor in which classes, relations, axioms, and instances can be modeled for building knowledge-based applications. The developed ontology was populated with 200 instances, and then exported in OWL. A series of reasoning procedures were carried out to validate the ontology, as presented in Section 4.2. In line with W3C best practices, the ontology was published online through GitHub [51], where it can be retrieved in a variety of serializations. In addition, diagrams, descriptions, human-readable documentation, and usage examples are provided to assist practitioners in familiarizing themselves with the ontology. The GitHub issues section provides a feedback and maintenance mechanism, allowing the continuous update and improvement of the ontology as needed.

### 3.3. RDF Processing Module

**Figure 6:**
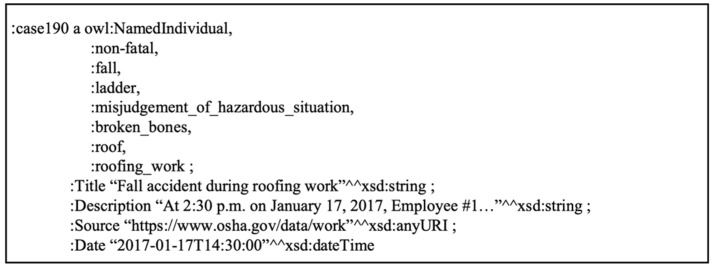
Accident case in the Turtle serialization.

The RDF processing module was developed to create a mechanism for the conversion of existing accident data and input of new data as RDF. In addition, the module provides users an environment to interact with machine-readable accident data in a variety of serializations. Initially, a Python-based virtual environment was initialized and the RDFLib Python library was imported along with its constituent functions. RDFLib is a Python library specifically designed for working with RDF data as graphs in a variety of serializations. By calling the graph function, the OWL database was parsed, and then serialized in the Terse RDF Triple Language (Turtle). Turtle is a text-based file format for expressing RDF data. It is easy to create and is more human-readable than syntaxes such as rdf/xml, json-LD, N-triples. Figure 6 presents an example of an accident case serialized in Turtle. As illustrated, each line is divided into a subject, predicate, and object. Case represents the subject/node, “a” represents rdf:type. Each case also has asserted data properties including a title, date, description, and source.

**Figure 7:**
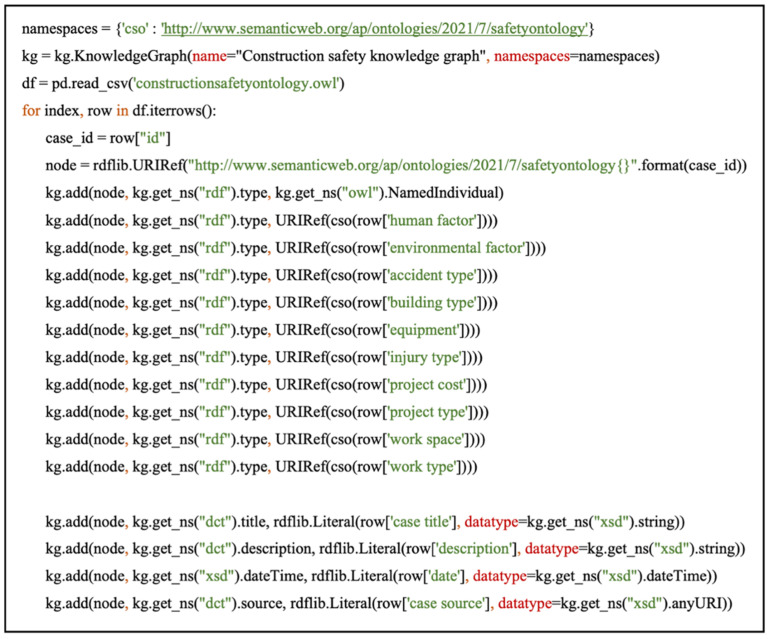
Algorithm for automatic conversion of accident data to RDF.

An algorithm utilizing the open-source KGLAB package was developed in Python for the conversion of existing accident data to RDF. The KGLAB package provides a simple abstraction layer for graph-based data engineering tasks. Using the Pandas package, a CSV file containing accident cases was read as a data frame. As portrayed in Figure 7, a program was written to iterate over each row of the data frame and (1) retrieve the accident case ID; (2) create a node with a unique URIRef by binding the case ID to the graph URIRef; (3) define the node as an OWL named individual (an instance); (4) add triples for each class and subclass designation, with the node as the subject, rdf.type from the rdf namespace as the property, and the object is called directly from the relevant row in the data frame; (5) similarly, triples are created for all the data properties. For the input of new accident data, a similar approach was deployed. Rather than accessing a data frame, a function was created to take in the accident case details based on the defined data schema. Using the developed iterative program, the details are then automatically added as triples into the dataset.

## 4. Results

To demonstrate the proposed concept and its technical feasibility, this section presents exemplary query- and graph-based visualization scenarios. Subsequently, validation procedures for the system modules are discussed.

### 4.1. Query Results

The query module utilizes SPARQL queries to allow users to retrieve safety information. SPARQL is the standard protocol designed and endorsed by W3C for querying linked open data and RDF datasets (triplestores). SPARQL queries are advantageous over conventional search approaches in that they are not constrained to local, isolated databases. Moreover, since SPARQL is an HTTP-based transport protocol, federated queries can access multiple data endpoints.

This section presents three query scenarios to demonstrate the effectiveness of the developed system in accessing information to acquire data-driven insights in safety management processes. The queries were executed in a Python-based virtual environment. The graph function of RDFLib was imported, and then used to parse the dataset as an RDF graph. Using RDFLib’s namespace manager, the prefix cso (construction safety ontology) was bound to the URIRef for the developed accident data graph. This made it easier to construct queries without having to repeatedly use a long URIRef.

**Figure 8:**
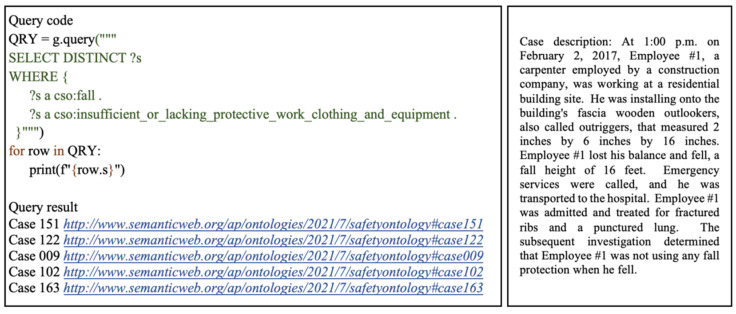
Query 1 code, result, and example of retrieved case.

**Figure 9:**
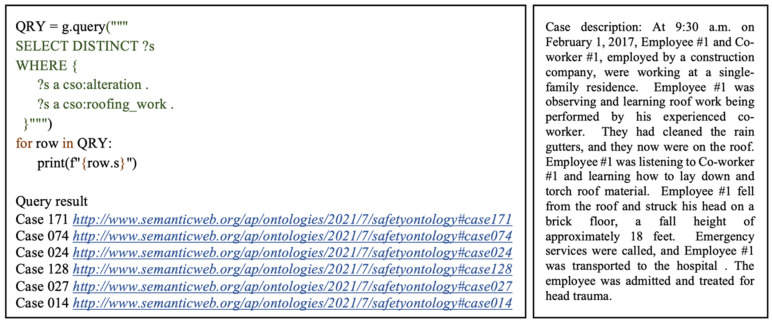
Query 2 code, result, and example of retrieved case.

The first query shows how related cases can be retrieved based on a specified project type and work type. This example considers a scenario searching for information on accidents which occurred during roofing alteration works. As illustrated in Figure 8, the SELECT query form is used to return all variables, or their subsets bound in the query pattern match. The DISTINCT modifier is also applied to eliminate any duplicated results that may bind the same variables to the same RDF terms. The variable ?s refers to all cases of interest which match the defined query parameters. The WHERE clause is used to specify the pattern to match against the data graph. In this case, the query pattern consists of two triples with the subjects as the case variable ?s, the predicate which represents rdf:type, and the objects are the IRIs for alterations and roofing works. This query pattern specifies that returned cases should belong to the alterations class, under the project type class, and the roofing works class, under the work type class. As illustrated in Figure 9, this query returned six cases which satisfied the specified conditions. By analyzing these cases, a user can acquire relevant related information and implement appropriate preventative measures in ongoing safety management processes.

The second query considers a scenario in which a safety practitioner searches for fall accidents which occurred due to insufficient or lacking personal protective clothing and equipment. This query aggregates all cases that belong to the fall subclass of accident type, and the insufficient_or_lacking_protective_work_clothing_and_equipment subclass of human factors under accident causes. Using the retrieved cases, a user can conveniently extrapolate existing trends and patterns in accidents of the specified type. For instance, during which work tasks and in which workspaces are such accidents prominent? In what environments are the consequences of such accidents more severe? Such data-driven lessons learned and insights serve as leading indicators to proactively prevent similar accidents from occurring repeatedly in the future. As depicted in Figure 9, out of the 200 cases in the RDF graph, five cases matched the user-defined query parameters.

**Figure 10:**
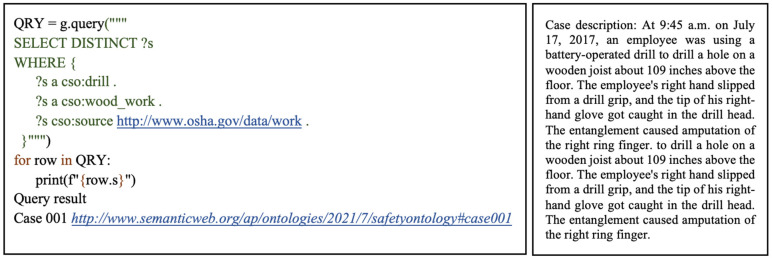
Query 3 code, result, and example of retrieved case.

The third query demonstrates how practitioners can execute highly faceted queries, to retrieve cases related to a particular work type, using specific tools and machinery. In this example, a user can search for cases involving woodwork with drills. Using the SPARQL WHERE clause, a graph pattern is specified to return cases that belong to the drill subclass of tools and machinery, under the equipment class, and the woodwork subclass of work type. In addition, this query illustrates how search results can be further narrowed down to return cases from a specific source. As depicted in Figure 10, this is executed through the third triple pattern under the WHERE clause, which specifies the predicate as cso:source and the specific source as the OSHA URL. This query returned one case which matched the user-specified parameters. The information retrieved from such a query could help in safety planning processes, making them more efficient. For example, a safety manager conducting a JHA could access cases related to the work type being considered, and acquire specific cases related to the equipment required for the work tasks. Insights from these cases would enable mitigation from the planning stages. Furthermore, the lessons learned from these past incidents can be disseminated to frontline personnel in safety training.

**Figure 11:**
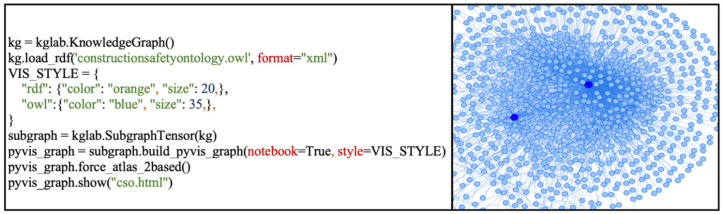
Graph-based visualization of accident data.

In addition to the developed query functionalities, this study also pioneers and explores graph-based visualizations of safety information through the KGLAB package for building knowledge graphs. Figure 11 presents a Python script and the corresponding graph-based visualization of the entire accident dataset. While this is an initial implementation with limited features and interactions, it is evident that graph-based approaches hold immense potential for data analytics in construction safety management. Rather than conventional tabular displays, graph-based approaches can provide a more intuitive and visually engaging representation of accident data and the relationships between elements in the data. Similarly query parameters and results can be visualized with a graph-based approach, allowing users to directly perceive the links and connections in the data.

### 4.2. Validation

**Figure 12:**
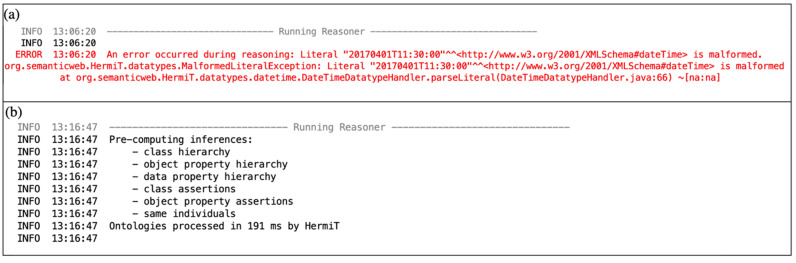
(**a**) Initial reasoning result with an error. (**b**) Subsequent reasoning result.

Firstly, in order to verify the consistency of the ontology module, Protégé’s inbuilt HermiT reasoner was deployed [52]. HermiT is an open-source reasoner designed specifically for ontologies written in the Web Ontology Language (OWL). Running the reasoner confirmed the structural and relational consistency of the developed ontology. Moreover, it assisted the authors in identifying and correcting instances where entered values were not valid for specified data types. Figure 12a presents the initial reasoning results, which yielded an error due to an entered value being malformed. Figure 12b presents the subsequent reasoning results after the formatting issue was resolved.

Secondly, SPARQL queries were executed in a Python-based virtual environment with a 2.3 GHz 8-core Intel Core i9 MacBook Pro. The Python timeit method was applied to measure the execution time for each query. As depicted in Table 2, each query accurately retrieved related cases which precisely matched the user-defined parameters in less than 2 s, confirming the functioning of the linked data system and its constituent modules.

Lastly, the W3C Shapes Constraint Language (SHACL) was used to validate the RDF graph against a set of conditions. SHACL requirements can be modeled as shape graphs, and the RDF graphs validated against the shapes are referred to as data graphs. A preliminary SHACL-based validation was conducted to verify that all accident cases in the graph were of the correct type, and had data properties of the appropriate datatype. The SHACL validation was executed by using the KGLAB package with Python. After instantiating a knowledge graph, a shape graph was defined. The prefixes for the required namespaces were specified, then the cso:caseshape SHACL shape was created, and defined as a node shape (sh:NodeShape). Since all cases in the data graph are instances of the type named individual, the target class (sh:targetClass) for the shape was set to owl:NamedIndividual. SHACL constraints were also defined to access the cso:date, cso:description, and cso:source data properties through sh:path, to confirm that cases in the dataset had data properties corresponding with the appropriate datatypes (xsd:datetime, xsd:anyURI, and xsd:string). Results revealed that all the accident cases in the data graph conformed with the validation criteria.

## 5. Discussion

This research presents a novel data-driven system which leverages linked data, ontologies, and knowledge graph technologies to enhance construction safety information sharing. The main contribution of this study lies in the technical modalities developed to resolve information dissemination challenges which past scholarly works and industry efforts have failed to address. The system modules have been validated using reasoning and SHACL shapes, and results from the initial evaluation confirm the effectiveness, accuracy, and efficiency of the approach in information retrieval. While initial findings are promising, there are several considerations warranting further attention in future research.

Firstly, the developed ontology was populated with instances manually in the current study. In addition to the pre-defined categories in the utilized OSHA dataset, several new categories were required to improve the query capabilities with the system. These included “equipment”, “work type”, and “workspace”. Manually sifting through hundreds of accident case descriptions to extract and input required categorical information tends to be not only time-consuming and inefficient, but also error prone. Hence, in this paper, additional data verification measures through ontology reasoning and SHACL constraints were required to ascertain the validity of data inputs. It would be worthwhile for future efforts to automate such laborious manual processes by drawing on advances in data science and natural language processing. This would in turn afford larger scale data-driven insights and predictive analytics from past accident information, which would enrich safety management processes.

Secondly, case studies are necessary to explore how the proposed system can be integrated and embedded into existing safety management tools and workflows. For instance, investigating how the developed solution can enhance BIM-based safety management operations would be a key consideration. Furthermore, in addition to the presented technical developments in this paper, the organizational implications of the approach need consideration. Additionally, further feedback from industry practitioners would be necessary to provide more insights into the practical applicability of the system in safety planning, training, and management scenarios. While this study presents a solution which contributes to effective sharing of safety information from past incidents, accident reports typically focus on downstream measures. It would also be worthwhile for future research to explore feasible approaches to not only capture, but also share, information pertaining to upstream measures such as safety culture and organizational safety climate.

Thirdly, while the presented solution facilitates safety information dissemination, system users need to be familiar with SPARQL to execute advanced information retrieval tasks. As part of the full system development, an end-user interface which takes query parameters in normal language and converts them to SPARQL will be required to make the system more accessible and readily usable for construction personnel. Lastly, the presented system implementation utilized one dataset which has not been published on a web server. It would be necessary for future works to verify the effectiveness of the proposed system in synthesizing information from diverse sources.

## 6. Conclusions

Accidents, injuries, and fatalities occur repeatedly in construction. Information from past mishaps is of tremendous value in transferring lessons learned and proactively preventing incidents. However, in practice, such information remains siloed and inaccessible due to a plethora of structural and organizational barriers. To understand the contemporary issues in accident information utilization, a thorough analysis of accident data was conducted, revealing several issues. Major impediments to safety information sharing include inconsistent data formats and storage methods across organizations, unstructured non-machine-readable data, lack of standardized parameters for accident data representations, lack of detailed categorical information, and limited detailed information regarding the circumstances around accidents.

To address these issues and enable the effective sharing of construction safety information, this study has developed a novel data-driven linked data system comprising three modules. Firstly, an ontology module was developed to formalize the expertise and knowledge in safety reporting, to establish a consistent structure in accident data representations, and ensure the quality and richness of incident descriptions as well. The ontology was populated with 200 accident cases using the Protégé authoring program. Secondly, an RDF processing module was developed to facilitate the automatic conversion of newly input data and existing tabular accident data into RDF graphs to enable information retrieval and reusability. This module leveraged state-of-the-art knowledge graph packages and libraries to develop conversion algorithms. Lastly, a query module was developed, deploying the SPARQL Protocol to facilitate query and information retrieval functionalities. In addition, the KGLAB package was utilized to enable novel interactions with accident data through graph-based visualization. A series of queries were executed to address the effectiveness and efficiency of the system in improving information retrieval and reusability. SPARQL queries with the developed system were found to yield highly specific and accurate data-driven results, which enhance safety management processes by making relevant lessons learned and insights readily available.

Considering the long-standing pervasiveness of accidents, injuries, and fatalities in construction, multi-faceted interventions are necessary to improve safety performance outcomes in industry. This paper presents the development of a data-driven solution for sharing construction safety information through linked data, ontologies, and knowledge graph technologies to embed lessons learned from past cases into safety management operations. The proposed system is envisioned to aid practitioners in conveniently acquiring insightful and relevant information during safety planning, training, and management processes, which would in turn contribute to proactive accident prevention. The deployment of the proposed approach introduces the possibility of a new “open” paradigm in safety information dissemination, with major implications for industry 4.0 and next-generation data-driven applications in construction safety management.

## Figures and Tables

**Table 1:** Prominent accident causation factors in construction based on data analysis.

	Accident Cause	Cases	Percentage
Human Factors	Misjudgment of hazardous situation	134	29%
	Inappropriate material/equipment handling method or procedure	75	16%
	Position inappropriate for task	25	6%
	Safety devices removed or inoperable	25	6%
	Insufficient or lacking protective work clothing/equipment	17	4%
	Insufficient or lacking written work practice program	17	4%
Environmental Factors	Work-surface or facility layout conditions	102	22%
	Overhead moving and falling object action	35	8%
	Weather-, earthquake-, nature-related	10	2%
	Pinch point action	9	2%
	Temperature tolerance levels	9	2%
	Sheer point action	8	2%

**Table 2:** Result accuracy and execution time for SPARQL queries.

SPARQL Queries	Result Accuracy	Execution Time
Query 1	100%	0.84
Query 2	100%	1.27
Query 3	100%	0.49

## Data Availability

The data presented in this study is available on request from the corresponding author.
